# *Pooideae* molecular sensitization profile in selected rhinitis patients with allergy to grass pollen and maize-derived edible products

**DOI:** 10.1186/2045-7022-5-S4-P21

**Published:** 2015-06-26

**Authors:** Florin-Dan Popescu, Mariana Vieru

**Affiliations:** 1 “Carol Davila” University of Medicine and Pharmacy, Department of Allergology, Bucharest, Romania

## Background

Clinical cases of allergic reactions induced by ingestion of maize-derived products have been rarely published, and most patients with food allergy to *Zea mays*, from the *Panicoideae* subfamily, are also sensitized against pollen of the *Pooideae* subfamily grasses, belonging to the same *Poaceae* family.

## Methods

Patients from Southern Romania with grass pollen rhinitis, diagnosed by allergy evaluation with positive skin prick tests and high serum levels of specific IgE to *Pooideae* pollen, were screened for food allergy by detailed clinical history of symptoms related to maize-derived edible products, including foods with corn as basic ingredient, such as polenta, porridge, cornflakes, tortilla chips, popcorn, canned kernels, or traditional products, such as stigmas and corncob leaves with pollen deposition. Patients with occupational exposure to maize flour or pollen were not included, and ceremonial use of oral corn pollen in the Romanian Plain was not reported. We assessed the IgE sensitization profile to pollen allergen components used as biomarkers of *Pooideae* allergy by a novel multiparameter immunoblot test system.

## Results

Two adult allergic rhinitis patients were selected, one woman with history of oral allergy syndrome to polenta and tortilla chips, and a man with urticaria after ingesting *Stigma maydis* infusion. Regarding the group 1 beta-expansins, with role in pollen tube penetration, and the group 12 profilin panallergens, important allergen components present in kernels and pollen, both patients had high levels of serum specific IgE to rPhl p 1, revealing true sensitization to *Pooideae* pollen, without IgE sensitization to rPhl p12. Only the patient with urticaria to corn silk had high level of specific IgE against polcalcin rPhl p 7, calcium-binding protein (CBP) likely to cross-react with Zea m 7 from maize pollen. IgE sensitization to the ribonuclease rPhl p 5 was not detected.

## Conclusion

Some pollen grass protein allergen components have good thermostability, maize polcalcin being probably involved in allergy to infusion of herbal products containing stigmas receiving pollen, while profilins possibly not, being more sensitive to heat denaturation. Maize beta-expansin 11, a Phl p 1 homologue, may have a high cross-reactive potential in patients who suffer both from food allergy and grass pollinosis. Interestingly, patients were not sensitized to grass pollen group 5 allergens, molecular components not representative in corn pollen.

## Consent

Written informed consent was obtained from the patient for publication of this abstract and any accompanying images.

